# Disparities in Infectious Diseases among Women in Developing Countries^1^

**DOI:** 10.3201/eid1011.040624_12

**Published:** 2004-11

**Authors:** Jamila Rashid, Olufemi O. Taiwo, Indu Ahluwalia, Stella Chungong

**Affiliations:** * Centers for Disease Control and Prevention, Atlanta, Georgia, USA;; † Somolu, and Bariga Local Government, Lagos, Nigeria;; ‡ World Health Organization, Geneva, Switzerland

**Keywords:** infectious disease, disparities, developing countries, conference summary

Infectious diseases are leading causes of death in developing countries. According to 1998 data from the World Health Organization, 1.2% of total deaths from infectious and parasitic diseases occurred in industrialized countries, compared to 43% in developing countries. Women in developing countries disproportionately suffer from a host of infectious diseases, including tuberculosis, upper respiratory tract infectious, skin infections, visceral leishmaniasis, malaria, sexually transmitted infections, and HIV/AIDS.

Poverty, poor education, low health knowledge, poor infrastructure, geographic factors, life style, and environmental factors (i.e., limited access to resources such as clean water) have been identified as primary factors contributing to the high incidence of infectious diseases among women in developing countries. Also, such women tend to have limited or no access to health care, be vitamin deficient, and have lower status in their communities.

Often, infectious diseases may run a substantially longer course for women in developing countries because of stigma, family needs, and shame. Another important barrier to combating infectious diseases among women in developing countries is the lack of use and the misuse of prescription medication. A substantial percentage of women rely on traditional treatment methods. When women do seek healthcare services and are prescribed medication, women frequently do not adhere to the dosage and quantities prescribed.

